# Lipoarabinomannan as a Point-of-Care Assay for Diagnosis of Tuberculosis: How Far Are We to Use It?

**DOI:** 10.3389/fmicb.2021.638047

**Published:** 2021-04-15

**Authors:** Julio Flores, Juan Carlos Cancino, Leslie Chavez-Galan

**Affiliations:** ^1^Laboratory of Integrative Immunology, Instituto Nacional de Enfermedades Respiratorias Ismael Cosio Villegas, Mexico City, Mexico; ^2^Laboratory of Immunomicrobiology, Department of Microbiology, Escuela Nacional de Ciencias Biológicas, Instituto Politécnico Nacional, Mexico City, Mexico

**Keywords:** lipoarabinomannan, tuberculosis, point-of-care assay, immune response, immunoregulation

## Abstract

Tuberculosis (TB) is still a severe public health problem; the current diagnostic tests have limitations that delay treatment onset. Lipoarabinomannan (LAM) is a glycolipid that is a component of the cell wall of the bacillus *Mycobacterium tuberculosis*, the etiologic agent of TB. This glycolipid is excreted as a soluble form in urine. The World Health Organization has established that the design of new TB diagnostic methods is one of the priorities within the EndTB Strategy. LAM has been suggested as a biomarker to develop diagnostic tests based on its identification in urine, and it is one of the most prominent candidates to develop point-of-care diagnostic test because urine samples can be easily collected. Moreover, LAM can regulate the immune response in the host and can be found in the serum of TB patients, where it probably affects a wide variety of host cell populations, consequently influencing the quality of both innate and adaptive immune responses during TB infection. Here, we revised the evidence that supports that LAM could be used as a tool for the development of new point-of-care tests for TB diagnosis, and we discussed the mechanisms that could contribute to the low sensitivity of diagnostic testing.

## Introduction

Tuberculosis (TB) is an infectious disease caused by the bacillus *Mycobacterium tuberculosis* (Mtb). Historically it has also been known as scrofula, Pott’s disease, King’s Evil, phthisis, and consumption, among others (Barberis et al., 2017). Currently, TB is a significant public health problem; the last report of the World Health Organization (WHO) in 2019 informed there were 10 million people with TB worldwide. Older men and women represented nearly 88% of all cases (World Health Organization, 2020). Risk factors such as diabetes mellitus, human immunodeficiency virus (HIV) co-infection, malnutrition, smoking, and alcoholism are associated with the development of TB (Glaziou et al., 2018; Melsew et al., 2018). The EndTB global project proposes that the design of new diagnosis methods or the improvement of the current diagnostic tests are a priority to accelerate the efforts to stop TB (World Health Organization, 2015b).

Mtb is an intracellular pathogen highly adapted to humans, which seems to have developed several mechanisms to avoid the host immune response and to persist indefinitely in the organism (Carranza and Chavez-Galan, 2019). The biological success of Mtb is due to critical features that have provided an evolutionary advantage, such as the loss of the coding region of the TbD1 gene and resistance to oxidative stress and hypoxia (Bottai et al., 2020).

Components of the Mtb structure are a source of antigens. For instance, the *Mycobacterium* cell wall has integrated virulence factors that are upregulated and are helpful in avoiding the host immune response (Bah et al., 2020). Lipoarabinomannan (LAM) is a cell wall glycolipid; its structural core is inserted into the plasma membrane of Mtb. LAM is considered as a potential tool to be used as a biomarker for TB diagnosis. Currently, LAM testing has some sensitivity-related disadvantages related to detecting and monitoring this antigen in urine and in peripheral blood.

**Figure 1:**
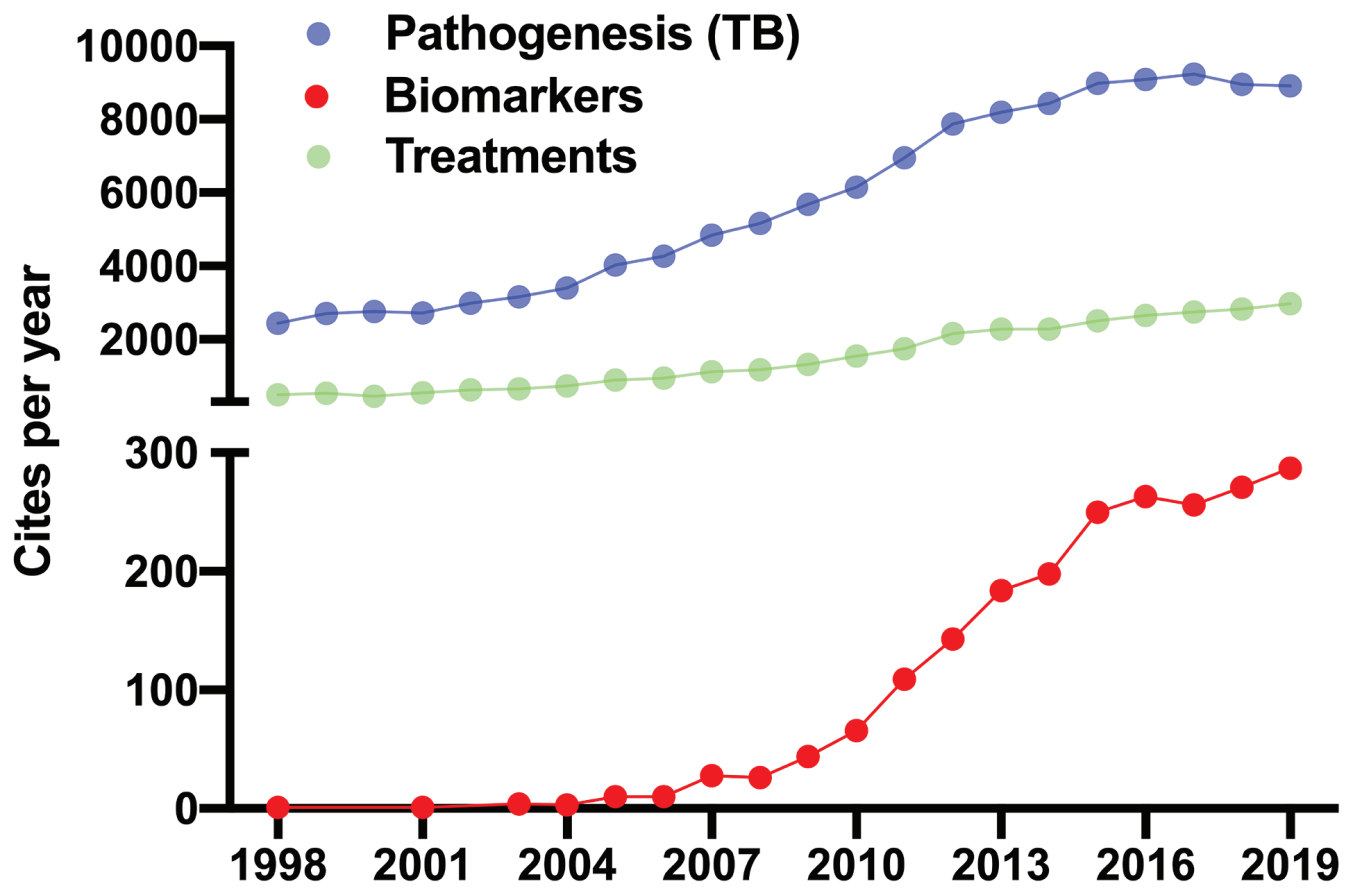
Recent approaches in tuberculosis research. Up to 2019, there were 162,356 cites in the tuberculosis (TB) field. 159,109 (98%) were focused on mechanisms of pathogenicity (purple dots) and generation of new drugs for treatments (green dots; left *y*-axis). Whereas 3,247 (1.3%) cites were related with studies exploiting the biomarkers field (red dots). Studies of LAM as a potential exclusive marker in the diagnosis of TB first appeared in 1998 and have increased ever since. The data was generated in Web of Science.

The publication of the Mtb genome sequence in 1998 was fundamental for a better understanding of TB pathogenesis (Cole et al., 1998), thenceforth, publications about TB have increased. Currently, papers related to pathogenesis and biomarkers are leading topics in the TB field, whereas the issue of TB treatments is the third most relevant (Figure 1). In this review, we discuss the role of LAM in the regulation of the host immune response during Mtb infection. Furthermore, the experimental evidence suggests that LAM could be used as a tool in the development of a new point-of-care test for TB diagnosis. Finally, we discuss the mechanisms that contribute to the low sensitivity of the LAM test.

## LAM: Origin and Structure

The processes of biosynthesis and metabolism of Mtb lipids remain poorly understood. Tuberculous and nontuberculous mycobacteria species have highly regulated genes associated with the metabolism/transport of lipids, and gene coding for proteins that modulate the cell wall structure (Fedrizzi et al., 2017; Tucci et al., 2020). Reports indicate that mycobacterial species have different types of regulation of the complex array of lipids, which provide significant differences in both the architecture and fluidity of the membranes (Adhyapak et al., 2020). During Mtb infection, lipid (including LAM) regulation is related to the modification of the physical properties of host cell membranes through the incorporation and diffusion of Mtb lipids (Raghunandanan et al., 2019; Mishra et al., 2020).

**Figure 2:**
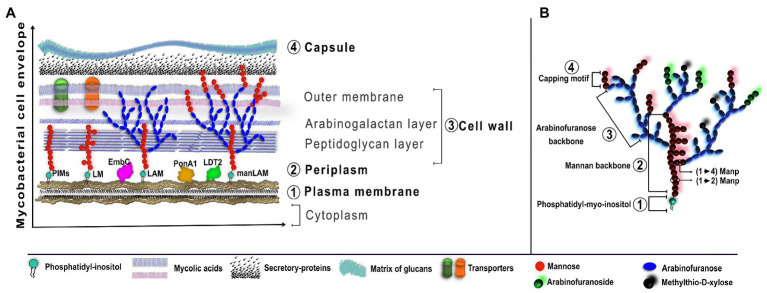
Schematic organization of LAM in the Mycobacterial cell envelope. **(A)** The cell envelope of Mtb is characterized by four basic regions, from the inner to the outer: plasma membrane, periplasm, cell wall, and capsule. The plasma membrane has anchored PIMs which are the basic structure for LM, LAM, and manLAM. Other assembling proteins, such as EmbC, PonA1, or LDT2, are required for the elongation process of LAM. **(B)** LAM from virulent Mtb is characterized by a four-domain structure: the phosphatidyl-myoinositol is anchored to the plasma membrane and is the foundation for the mannan backbone, followed by the arabinofuranose backbone and the capping motifs of extra-mannoses, methylthio-D-xylose, or arabinofuranosides. The figure was created in affinity designer.

The mycobacterial cell envelope has proteins and lipids distributed in four layers; such layers are unique regarding shape and chemical characteristics. The spatial arrangement, from the inner to the outer layers, is: (1) the plasma membrane, which has glycolipids derived from phosphatidylinositol (PI) with different forms of mannosylation and acylation, such as phosphatidyl-myo-inositol mannosides (PIM), lipomannan (LM), and LAM; (2) the periplasm, which includes assembling proteins such as EmbC, PonA1, or LDT2; (3) the cell wall, which consists of a layer of peptidoglycan and arabinogalactan, and an outer membrane composed of mycolic acids like trehalose monomycolates, and porins; and finally; and (4) the capsule, which is a matrix of glucans and secretory proteins such as CFP-10 and ESAT-6 (Figure 2A; Chiaradia et al., 2017; Dulberger et al., 2020).

Virulent strains (such as H37Rv, Erdman, or NYH-27), avirulent strains, or clinical isolates of Mtb show differences in the LAM structure, even compared to other pathogenic mycobacteria like *M. smegmatis* or *M. leprae* (Chatterjee et al., 1992; Choudhary et al., 2018). The variability in the branches of LAM structure is determined mainly by the following aspects: (1) type of molecule attached in the terminal ends, meaning that mannose, arabinose, or methylthio-D-xylose (MTX) could induce a more or less complex structure; and(2) structural configuration of epitopes, wherein modifications such as (Manp)1 or 2-linked branches, chains of four or six arabinoses, and MTX-linked to Man branches are central to determining the length and composition of the epitope (Magni et al., 2020). It has been observed that these structural components of LAM are dynamic and have implications regarding epitope recognition. For instance, reports have demonstrated that the ability of specific epitope recognition of some antibodies (CS-35, A194, MoAb1, S4-20, FIND 28, CS40, and CS906.7) is modified due to the changes in the LAM structure (Yang et al., 2013; Choudhary et al., 2018; Sigal et al., 2018; Magni et al., 2020).

The main feature of LAM derived from virulent Mtb is the presence of extra mannose caps, which is called manLAM. Hereafter, when we use “manLAM,” we are referring to LAM from Mtb virulent strains. Thus, LAM and LM show two principal structural domains: (1) a core of PIM, which is anchored to the inner membrane by non-covalent binding and, (2) a mannan backbone bound to the core of PIM, constituted by mannose groups such as α-(1→2) or α-(1→6) manno pyranose (Manp)-linked branches. In the specific case of manLAM, it has a terminal cap called “capping motif of LAM”; these terminal residues could be mannose or tetra-/hex-arabinofuranoside (Figure 2B; Angala et al., 2017; Abrahams and Besra, 2018; Choudhary et al., 2018).

The reactions of mannosylation (enzymes such as PimA, PimB, and PimE) and acetylation (enzymes such as PatA) are carried out in the core of PIM structure as a first step in the LAM and LM synthesis in the cytoplasm of Mtb (Korduláková et al., 2002, 2003; Lea-Smith et al., 2008). Posteriorly, these PIM forms (acetylated and mannosylated) are exported by the ATP-binding cassette (ABC) transporter, from the cytoplasm to the plasma membrane or periplasm (Glass et al., 2017). Here, a group of mannosyltransferases enzymes (such as MptA, MptB, MptC, and, MptD) add long mannose chains with α-(1→2) and α-(1→6) linkage (Mishra et al., 2011; Cashmore et al., 2017). Subsequently, to obtain the standard LAM structure, there are arabinofuranosyltransferases (such as AftA, AftC, AftD, or EmbC) to form α-(1→5) and β-(1→2) linkages and to add arabinofuranose residues (Shi et al., 2006; Jankute et al., 2017; Angala and Jackson, 2019; Tan et al., 2020).

To obtain the final manLAM structure, there is extra and random formation of capped end residues. These are groups of one, two, or three mannoses in α-(1→5) and α-(1→2) bonds mediated by ManpT and MptC enzymes, respectively (Dinadayala et al., 2006; Kaur et al., 2008). Another residue described is a MTX in α-(1→4) bond (Turnbull et al., 2004; Angala et al., 2017). Reports suggest that modifications in some of these enzymes give rise to differences in the elongation of LAM and LM, generating truncated structures that decrease LAM formation and change the PIM/mannose/arabinose ratio. Whereas wild strains have a 1:100:7 ratio (PIM/mannose/arabinose, respectively), the modified strains have 1:30:0.13 (Alonso et al., 2017; Cashmore et al., 2017).

PI is necessary for the basic LAM structure, so that LAM can be attached to the plasma membrane (Källenius et al., 2016; Cashmore et al., 2017). Enzymes such as cytidine diphosphate alcohol phosphotransferase and phosphatidylinositol phosphate synthase affect the PI structure and, consequently, the remodeling process of the cell envelope is modified (Clarke et al., 2015; Sohlenkamp and Geiger, 2016; Belcher Dufrisne et al., 2020). LAM can also arise from modified forms of PI, and these forms can activate the immune response mediated by receptors (Barnett and Kagan, 2020).

Mtb is highly able to adapt under stress conditions, and it can do proteomic and lipidomic rearrangements; in this way, reports indicate that the distribution of glycolipids, including LAM: LM ratio, is affected during the different stages of infection (Eoh et al., 2017; Baker and Abramovitch, 2018; Garcia-Vilanova et al., 2019; Vinhaes et al., 2019). *In vivo* and *in vitro* models have demonstrated that low LAM concentration can induce a negative regulation of the activation of the immune system (Driss et al., 2012; Chávez-Galán et al., 2015; Yuan et al., 2019). Perhaps this is because LAM possess multiple epitopes, and these interact with cell surface receptors in different grades of affinity (Choudhary et al., 2018; De et al., 2020; Zhou et al., 2020).

Nearly 70% of LAM caps are mannoses. Studies suggest that under a status of reactivation, Mtb modifies the LAM structure through mechanisms such as the addition of succinyl groups to arabinomannan domains by the action of acyltransferases. SucT, MtxS, and MtxT glycosyltransferases, and the arabinofuranosyltransferase D (AftD), add inositol phosphate or methylthioxylose in the mannoside cap of manLAM (Oi et al., 1982; Khoo et al., 1995; Angala et al., 2017; Palčeková et al., 2019).

Thus, LAM immunogenicity is highly dependent on post-transcriptional modifications like random glycosylation patterns of mannose and arabinomannan motifs. Reports suggest that these post-transcriptional modifications increase the LAM antigenic capacity up to 10 times. For instance, free LAM is associated with a decrease of O-mannosylation and an increase of O-glycosylation (Alonso et al., 2017; Birhanu et al., 2019). These modifications in the LAM structure are essential to Mtb that develop adaptive processes, such as heterogeneous glycoproteomic patterns that can increase the current phenotypic variability.

There is still a long way to obtain the complete structure of native LAM. This is in part because both extraction and purification processes are unspecific (Rahlwes et al., 2019). Recent studies have demonstrated that glycoconjugate synthesis could be an alternative method to study the structural and physical characteristics of LAM (Zhang et al., 2018; Chang et al., 2019). In this context, the generation of synthetic molecules makes the study of LAM as an Mtb virulence factor more accessible (Gao et al., 2014).

Thus, the knowledge of the LAM structure facilitates the understanding of how the components of the mycobacterial cell wall have immunological implications. For instance, as discussed below, LAM has the ability to modulate the host immune response but, interestingly, the induced changes could be different during each infection stage, which could be related to the modifications that LAM suffers during its synthesis.

## Immunoregulation Induced By LAM

The early stage of Mtb infection is characterized by an inflammatory response, which plays a protective role, whereas in advanced stages, the cell-mediated immune response is mainly immunosuppressive. LAM is recognized through mannose receptor (MR), dendritic cell-specific intercellular adhesion molecule-3-grabbing non-integrin (DC-SIGN), Toll-like receptors (TLRs), and C-type lectin receptor (Dectine-2; Wu et al., 2011; Yonekawa et al., 2014; Shukla et al., 2018).

*In vitro* studies have demonstrated that LAM has a different amount of mannose units, and that increased mannose concentration affects directly the maturation and function of dendritic cells (Zhang et al., 2019). The glycosyltransferases MtxS and MtxT promote discrete glycosyl substitutions in the mannoside cap of manLAM, which favors a structural microheterogeneity. Thus, it may induce an immunomodulatory effect in host cells (Angala et al., 2017). Through the use of synthetic mycobacterial glycan arrays, it has been possible to observe that LAM structural changes have different outcomes. On the one hand, if LAM has several glycan receptors overlapping, and pathway redundancy are favored, this would allow for mycobacteria to enter the cell. On the other hand, the consequence of this “easy” cell infection is an increased presence of potent immunogenicity inducers (Zheng et al., 2017; Zhang et al., 2018; Chang et al., 2019).

Reports indicate that LAM can be found in urine, serum, and lung from TB patients (Broger et al., 2019b; Brock et al., 2020; De et al., 2020). Recent clinical studies indicate that maternal milk could be an additional source of LAM in pregnant women (García et al., 2019; Pasipamire et al., 2020). Moreover, it is possible to observe variations in the profile of circulatory cell subpopulations, cell surface markers, and circulating soluble proteins in patients with latent (_L_TB), subclinical, or active (_A_TB) TB. The previous favors the hypothesis that if LAM is circulating in the peripheral blood, it could directly influence the leucocyte response and, consequently, LAM could be a potential tool for future vaccine or treatment design due to its relevant immunomodulatory effect. In this regard, it is not surprising that the presence of circulating LAM impacts on the activation of both innate and adaptative immune responses.

### Modulation of the Innate Immunity by LAM

**Figure 3:**
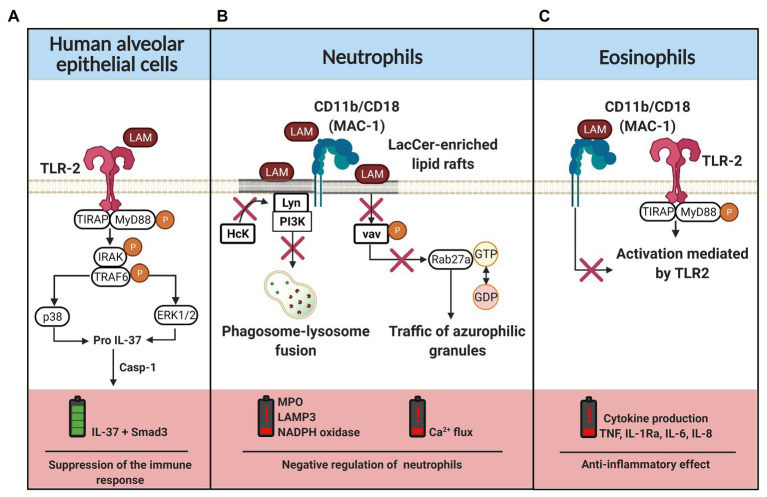
LAM-induced regulation of the innate immune cells. **(A)** Interaction of LAM with TLR-2 in human alveolar epithelial cells, the classical pathway of TLR activation is mediated by MyD88 and TIRAP phosphorylation. Downstream, IRAK and TRAF molecules are phosphorylated to induce p38 and ERK1/2 activation, which are involved in pro-IL-37 production. IL-37 maturation requires caspase 1 (casp-1), IL-37 forms a complex with Smad3 that is translocated into the nucleus to suppress the inflammatory response. **(B)** LAM is inserted into the lactosylceramide (LacCer)-enriched lipid rafts or it can be recognized by the CD11b/CD18 complex. When LAM is inserted in the cell membrane, the recruitment of hematopoietic cell kinase (Hck) by Lyn kinase is inhibited. Moreover, LAM regulates the phosphorylation of vav and, consequently, there is a downregulation of ras-related protein (Rab27a), limiting the traffic of azurophilic granules by GTP hydrolysis blockade. Thus, in neutrophils, LAM favors a negative regulation characterized by low production of myeloperoxidase (MPO), NADPH oxidase, LAMP3, and impaired intracellular calcium flux. **(C)** CD11b/CD18 expressed on the cell surface of eosinophils interact with LAM, blocking the TLR-2 mediated cell activation and consequently the inflammatory cytokine (TNF, IL-1Ra, IL-6, and IL-8) production is limited. The figure was created in BioRender.

Once Mtb arrives at the lower respiratory tract, its virulence factors (including LAM) combat the host defense system through mechanisms that affect the viability and functionality of innate immune cells such as alveolar epithelial cells, macrophages, and dendritic cells (Pavlicek et al., 2015; Palčeková et al., 2020; Rodrigues et al., 2020). In this regard, several pieces of evidence have revealed that LAM possesses heterogeneous immunogenicity that induces different signaling mechanisms. For instance, it has been reported that dendritic cells have a specific response to each level of the structural complexity of LAM (Zhang et al., 2019). Reports have demonstrated that LAM induces interleukin (IL)-37 production in human type II alveolar epithelial cells through TLR-2 signaling, p38 upregulation, and ERK1/2 phosphorylation (Huang et al., 2015; Figure 3A). Apparently, IL-37 precursor is converted to IL-37 by the action of caspase 1 (Casp1). IL-37 forms a complex with Smad3 (SMAD family member 3), and is translocated into the nucleus to inhibit the signal transduction of proinflammatory cytokines (Yan et al., 2019).

Recently, it has been described that IL-37 suppresses activation, proliferation, and cytokine production through down-regulation of nuclear factor-kappa B (NF-κB), and that it inhibits macrophage polarization into the M1 subtype (Zhou et al., 2020). However, this immunomodulatory effect is not observed in _A_TB patients, who have high circulating levels of IL-37 but not a high expression of its receptors IL-18R and IL-1Ra; this is probably a regulatory mechanism exclusive of the alveolar region (Wawrocki et al., 2019; Moideen et al., 2020; Zhang et al., 2020).

LAM can block the activation and differentiation of several cell subpopulations. *In vitro* evidence has shown that monocytes exposed to LAM are differentiated into immature macrophages; phenotypically these lack CD86, TLR2, and TLR4 expression. These macrophages have an impaired intracellular signal activation to tumor necrosis factor (TNF), and consequently PAR2 pathways are deficient in controlling intracellular Mtb growth (Chávez-Galán et al., 2015, 2017; Carranza and Chavez-Galan, 2019).

Neutrophil classical pathway activation includes signaling transductions near lipid rafts, which favor mobilization and activation of the Scr protein family. Subsequently, molecules such as MAPKs, Akt, PLC, and vav are phosphorylated. This increases the intracellular calcium flow, the reorganization of the cytoskeleton to induce phagocytosis, and the release of pro-inflammatory mediators such as elastase, PGE2 (prostaglandin E2), MMP-8 (matrix metalloproteinase-8), lysozyme, leukotriene B4, or TNF (Warren et al., 2017). Nakayama et al. (2016) have demonstrated that LAM is essential for neutrophils to engulf the mycobacteria through CD11b/CD18 molecules. LAM is associated with lactosylceramide-enriched lipid rafts localized on the neutrophil membrane to disrupt the signaling of the tyrosine-protein kinase, inhibiting the association of hematopoietic cell kinase (Hck) with Lyn tyrosine kinase to prevent phagolysosome formation, cytokine production, degranulation, and respiratory burst. Moreover, reports suggest that LAM recognition by neutrophils regulates the exocytosis of secretory vesicles (Miralda et al., 2020). Together, these results provide further support for the hypothesis that LAM insertion into lipid rafts regulates vav phosphorylation, consequently downregulating ras-related protein (Rab27a) and limiting the traffic of azurophilic granules by blocking the GTP hydrolysis (Figure 3B). Thus, these results highlight the role of LAM to inhibit the phagosome maturation and affect an adequate activation of the innate immune response.

TLR1 alleles such as 743A/1805G, 743AG/1805TG, and 743AG + GG/1805TG + TT have been associated with increased susceptibility to TB (Liang et al., 2017). Recently, it has been reported that neutrophils with the TLR1 1805G/T polymorphism are activated by LAM *via* TLR2/1 to produce pro-inflammatory cytokines; however, it is independent of the classical pathway induced by the synthetic ligand Pam3CSK4 (Hook et al., 2020). However, it is still unclear if the presence of polymorphisms modifies the affinity of TLR1 for LAM. It has been demonstrated that the absence of mutations in this receptor decreases TLR-mediated activation. Moreover, it has been suggested that there is self-regulation mediated by an integrin-type molecule or junk receptors that activate the E3 ubiquitin ligase Cbl-b (casitas B-lineage lymphoma proto-oncogene-b), which is responsible for MyD88 and TIRAP proteasomal degradation when TLR signaling is negatively regulated (Han et al., 2010).

Evidence shows that TB patients have circulating monocyte and neutrophil subpopulations related to a pro-inflammatory phenotype, and it has been suggested that those cells are more susceptible to apoptosis (Chávez-Galán et al., 2012; Lastrucci et al., 2015). This could be associated with the presence of circulating LAM in serum. In support of this hypothesis, it was recently reported that Mtb and its virulence factors (including LAM) interact with myeloid progenitors to induce impaired hematopoiesis (Khan et al., 2020). Moreover, it has been demonstrated that circulating LAM in serum from TB patients can be associated with high-density lipoprotein (HDL), and it has been suggested that HDL promotes mycobacterial infections in human macrophages with a remarkable decline in TNF production (Sakamuri et al., 2013; Inoue et al., 2018). The LAM/HDL ratio poses several questions; for instance, there is no evidence indicating that HDL allows the LAM insertion into the membrane of the host cells or that HDL promotes LAM entry to the host cell cytoplasm. It is important to elucidate if LAM and HDL must act together to induce alterations in the immune response, or if alterations are also induced when they act separately.

Although the contribution of eosinophils to the control of TB infection has been revised, the eosinophil-Mtb interaction remains poorly understood (Garg et al., 2017; Prakash Babu et al., 2019). Results observed by Driss et al. (2012), suggest that LAM has an eosinophil anti-inflammatory effect, as LAM down-regulate the release of peroxidase and TNF through the CD11b/CD18-LAM interaction, which limits the TLR-mediated activation (Figure 3C). In this regard, Niekamp et al. (2019) have provided evidence in support of Mtb-catabolic activity induction in the sphingolipid pathway, consequently remodeling lipid rafts during phagocytosis. Similarly, it has been suggested that LAM modulates the ceramide synthesis and alters the ERK-dependent signaling pathway (Sirkar and Majumdar, 2002).

Collectively, these observations suggest that LAM affects the phenotype and the functionality of the innate immunity cells; as a result, the activation of the innate immune response against Mtb is delayed or deficient. Additional studies have demonstrated the potential of many molecules that may promote the reactivation of cells exposed to LAM, such as high-mobility group box 1 (HMGB1), a danger-associated molecular pattern (DAMP) that can enhance the innate response to Mtb-antigens like LAM (Lui et al., 2016).

### Modulation of the Adaptative Immunity by LAM

Similar to the alterations observed in the innate immunity induced by LAM, several reports have demonstrated that the interaction between LAM and the cells of the adaptive immunity has negative implications in the response against Mtb.

**Figure 4:**
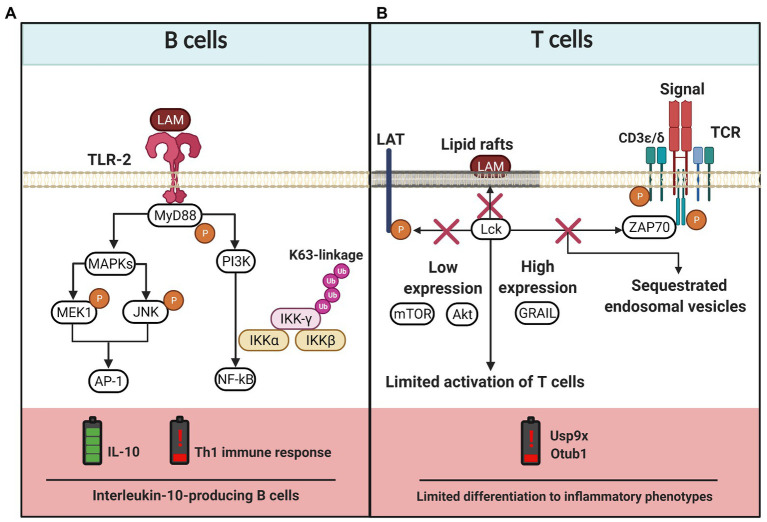
LAM-induced regulation of the adaptative immune cells. **(A)** B cells express TLR-2 that recognizes LAM and activates MyD88-dependent pathway. The TLR-2 signaling cascade activates the PI3K pathway and the MAP kinase (MAPKs) pathway, the latter induces MEK1 and JNK phosphorylation and activation of AP-1. While the PI3K pathway activates NF-kB, both pathways lead to IL-10 production. Moreover, TLR-2/LAM complex induces polyubiquitylation of IKK-γ (subunit of NF-κB essential modulator) by K63 activity and, consequently, the Th1 immune response is affected. **(B)** The LAM insertion into lipid rafts in T cells blocks the activation process since phosphorylation of lymphocyte-specific protein tyrosine kinase (Lck), linker for activation of T cells (LAT), Zeta-chain-associated protein kinase 70 (ZAP70), CD3-epsilon, and CD3-delta chains is inhibited. LAM also induces low expression of Akt and mTOR, whereas the levels of genes related to anergy in lymphocytes (GRAIL) increases, together limiting the T cell activation. Finally, there are also reports suggesting that LAM induces the limited phosphorylation of zeta chains that sequester endosomal vesicles and favor low expression of Usp9x and Otub1 enzymes, consequently limiting the differentiation to inflammatory phenotypes. The figure was created in BioRender.

In the context of Mtb infection, the presence of regulatory cytokines, such as IL-10, is harmful to the host. An IL-10 enriched microenvironment promotes Mtb survival (Wong et al., 2020). Evidence suggests that LAM promotes an IL-10-producing B cell (B-10) subpopulation (CD1d^low^CD5^+^) increase, which induces an immunomodulatory mechanism to inhibit the development of a pro-inflammatory environment against Mtb. Opposingly, IL-4^+^ Th2 polarization leads to an increased susceptibility to mycobacterial infection (Yuan et al., 2019). Thus, LAM is recognized by TLR2 expressed on B10 cells, favoring IL-10 production *via* AP-1. This pathway also regulates the NF-kB-mediated inflammatory gene transcription by K63-linked ubiquitination of NF-κB essential modulator (NEMO; Figure 4A).

T cell activation is also affected by LAM; *in vitro* methods made it possible to observe that LAM is inserted into CD4^+^ T cell membranes mainly in lipid raft sites. Here, LAM blocks the phosphorylation of molecules like Zeta-chain-associated protein kinase 70 (ZAP-70), ζ-chains of the CD3 (CD3 ζ), lymphocyte-specific protein tyrosine kinase (LcK), and linker for activation of T cells (LAT), which are indispensable to activate CD4^+^ T cells by the classical TCR-dependent pathway (Mahon et al., 2012). A deeper analysis has demonstrated that LAM induces hyporesponsiveness in CD4^+^ T cells through the upregulation of genes related to anergy in lymphocytes (GRAIL). LAM inhibits Akt and the mammalian target of rapamycin (mTOR) phosphorylation, and decreases the expression of deubiquitinating enzymes such as ubiquitin-specific protease (Usp9x) and ubiquitin thioesterase (Otub1). All these together result in impaired CD4^+^ T cell activity by LAM effect (Sande et al., 2016; Karim et al., 2017; Figure 4B). Athman et al. (2017) have demonstrated that Mtb-infected macrophages release Mtb-derived bacterial vesicles (BV) rich in glycolipids like LAM and LM. These BV are transferred to T cells, where the lipoglycans induce a GRAIL-dependent mechanism to inhibit T cell response.

The effect of LAM on cytotoxic (CD8^+^ T) cells has been less studied. Evidence has shown that LAM is one of the most potent mycobacterial lipid antigens that activate a lymphocyte subpopulation called polycytotoxic T cells, and a correlation between the frequency of polycytotoxic T cells and the ability to control infection has been described (Busch et al., 2016).

Although alterations in CD8^+^ T cells during TB have been reported (Busch et al., 2016; Chávez-Galán et al., 2019; Shen et al., 2019), there are still several questions about how to regulate the function or presence of these cells under a TB context. It is necessary to identify if LAM could be used to improve current immunotherapy against Mtb. As LAM reaches different cells and organs in TB patients through circulating peripheral blood, it is imperative to identify how LAM affects the functionality of the immune cells to delay an adequate response.

### Exploiting the Advantage of the Immunomodulatory Effect of LAM to Implement Therapeutic Alternatives

As we have discussed above, the immunomodulatory effect of LAM on the host cells is clear, and it suggests that LAM could be used in the field of therapeutic alternatives seeking. Focus has been put on two major paths: (1) generating a protective effect in people with a high risk of developing an inflammatory disease, or (2) modulating specific cells under the context of infectious diseases and cancer.

LAM expands regulatory cell subpopulations, such as B10 cells, which have a high capacity to decrease an inflammatory response; it has been reported that adoptive transfer of regulatory B cells induced by LAM reduces the severity of sustained systemic inflammation in the context of inflammatory bowel disease (Yuan et al., 2020). This opens new opportunities to generate customized therapy in humans.

LAM regulates specific signaling pathways, which have been used to eliminate visceral leishmaniosis infection in macrophages. In this context, reports have demonstrated that manLAM could upregulate the intracellular Ca^2+^ flux and the NOD2 receptor activity. The man-LAM-induced activation of infected macrophages enhances the production and delivery of IL-12, interferon-gamma (IFN-γ), and TNF; similarly, the frequency of cytotoxic T cells positive to IFN+ and CD4^+^IFN^+^ is also increased (Jawed et al., 2018; Roy et al., 2020). Altogether, these results highlight the importance of LAM in the clearance of intracellular parasites.

Finally, under the cancer context, it has been recently reported that tolerogenic dendritic cells (DCs) stimulated with LAM can activate tumor-specific CD8^+^ T cells. Moreover, these DCs were highly able to capture tumor antigens and to upregulate co-stimulatory molecules in order to prime naïve T cells and differentiate them into tumor-specific CD8^+^ T cells (Tomita et al., 2019).

## Assessment of LAM Tests for the TB Diagnosis

In parallel to the study of LAM as a molecule with the ability to regulate the immune response, and considering that even during a localized TB infection (for instance, pulmonary TB) LAM is found at the infection site (e.g., in circulating blood and urine), emerges the motion to incorporate LAM as a target molecule in the development of a novel point-of-care assay to improve TB diagnosis. Precise and timely diagnosis is essential to implement an effective and successful TB treatment. There are characteristics that TB diagnostic tests must guarantee, such as sensitivity, specificity, and quickness, as well as the ability to distinguish the TB pathophysiological spectrum. Additionally, it is imperative that the clinical specimen is easy to collect. A gold-standard diagnostic testing must be low-cost (as much as possible) and be easy to interpret.

TB diagnosis is complicated because some Mtb infected subjects develop _A_TB with clear symptoms and signs of the disease, whereas other patients do not display clinical evidence of disease even when they are infected (_A_TB and _L_TB, respectively). The presence of _L_TB also favors the spread of Mtb to other subjects and, consequently, the disease propagation is increased. Thus, a series of tests have been recommended to guarantee a correct TB diagnosis, which include interferon-gamma release assays (IGRA), tuberculin skin test (TST), acid-fast bacilli (AFB) smear, liquid and solid mycobacterial cultures, nucleic acid amplification test (NAAT), and biomarker-based assays, among others (Lewinsohn et al., 2017; World Health Organization, 2020). The rapid diagnosis of TB caused by multidrug-resistant Mtb strains is another emergency that requires a solution to reduce the mortality of TB; in this way, WHO recommends the use of Xpert tests, which is a NAAT test that detects drug resistance-related mutations in Mtb (World Health Organization, 2015a).

TB incidence is higher in developing countries. Unfortunately, to manage a wide variety of tests and to be able to make an appropriate diagnosis, both clinical and public health laboratories must have access to relatively sophisticated equipment. Consequently, the price of a diagnostic test is expensive. Moreover, the follow-up of patients should also be considered in order to confirm that the individuals are negative to Mtb infection or to discard the cytotoxic effect of the anti-TB drugs. Unfortunately, the economic aspect is one of the most important reasons for TB diagnosis delay.

A timely TB diagnosis is also necessary to interrupt TB transmission. Several _L_TB patients that lack a proper diagnosis will arrive at the hospital only when they show classic symptoms of _A_TB and, frequently, until clinical complications have developed. Currently, the need to identify new antigens for the development of vaccines, treatments, and diagnostic tests is worth noting. LAM has been discussed as a potential tool to design better testing, which should be able to distinguish the physiopathologic spectrum of TB patients (Drain et al., 2018; Correia-Neves et al., 2019).

### Advances in the Use of Urinary LAM Assay for TB Diagnosis

Hamasur et al. (2001) proposed for the first time the use of LAM as a biomarker for TB diagnosis. They reported the detection of urinary LAM through the enzyme-linked immunosorbent assay (ELISA). Apparently, during blood filtration in the kidney, the glomerular endothelial cells form a network that exhibits small orifices with a size enough to allow through Mtb-derived membrane molecules or extracellular vesicles carrying LAM, and consequently LAM is excreted in the urine (Dahiya et al., 2019).

Worldwide, the use of a rapid test for TB diagnosis is not broadly accepted (Thit et al., 2017; Bjerrum et al., 2019; Songkhla et al., 2019). Studies show that a disadvantage of the tests based in LAM creates the high amount of positive/negative-false results. A reason for this is the reduced specificity of anti-LAM antibodies, which induces cross-reactivity with nontuberculous mycobacteria (Dheda et al., 2010). In many cases, commercially available tests are using antibodies produced in small animal models. In contrast, it has been proposed that the development of antibodies in large animal models, such as llamas or goats, increase antibody titers and avidity, which is convenient for both improving the test sensitivity and saving money (Thompson et al., 2016). It could also be helpful to develop a more accessible test, in economic terms.

AlereLAM (Alere Determine TB LAM Ag) is one of the current point-of-care TB assays; it uses a methodological design of lateral flow assay and is the only one commercially available. One of the most critical advantages of AlereLAM is its price, which is 3 or 4 times lower than the nucleic acid amplification tests (Kerkhoff and Lawn, 2016). However, AlereLAM has suboptimal sensitivity. For instance, it is not recommended for LAM detection in patients with CD4^+^ T cell counts greater than 200 cells/mm^3^. AlereLAM has generated a large number of misinterpretations that depend on clinical characteristics such as CD4^+^ lymphocyte count, bacillary loads, or clinical symptoms that have been exposed in the WHO guidelines of 2019 (Bjerrum et al., 2019; Songkhla et al., 2019; World Health Organization, 2019). Unfortunately, AlereLAM has restricted use in clinical practice.

The objective of the development of the Fujifilm SILVAMP TB LAM test (FujiLAM) is to improve the sensitivity of AlereLAM (Broger et al., 2019a). Studies suggest that FujiLAM is a feasible technology for urine LAM detection with the potential to improve rapid diagnosis of TB at the point-of-care. FujiLAM has been recommended to be used in children, including hospitalized children with HIV-infection or malnutrition (Bulterys et al., 2019; Nicol et al., 2020). Recently, it has been demonstrated that the use of Xpert plus FujiLAM test for TB testing in hospitalized people with HIV is a cost-effective test compared with sequential testing and CD4-stratified testing strategies. Moreover, the implementation of the use of Xpert plus FujiLAM was related to increased life expectancy, possibly because it is helpful to obtain fast and accurate TB diagnosis (Reddy et al., 2020).

Another technology that emerged as a point-of-care (POC) platform for real-time evaluation of urinary LAM is the so-called photonic biosensors. The principle of this method is based on refractive index changes, which are present when the specific antibody (anti-LAM) is bound to the analyte. Then, the signal is measurable as a resonant wavelength shift through an interferometer and a chip-spectral (Martens et al., 2018). Evidence about the use of the POC platform is limited, but authors reported that this test provides results in a short time (15 min) and the detection limit of urine LAM is 475 pg/ml (Ramirez-Priego et al., 2018). As an alternative, a plasmonic fiber optic absorbance biosensor could detect LAM at ultra-low concentrations of a few fg/ml (Divagar et al., 2020). However, the POC platform has been used only in _A_TB patients, so it is necessary to include other groups within the TB spectrum to confirm the specificity and sensitivity of the POC platform for urine LAM detection.

As was discussed above, the Mtb metabolic activity changes during the infection period, which probably has a direct impact on the LAM structure, and consequently the sensitivity and specificity of the rapid tests are affected. It is well known that the ELISA test specificity is improved using monoclonal antibodies. In this way, the LAM-specific antibody should be selected carefully because it is probable that the variations between TB patients can be associated to the diverse modifications induced in the LAM structure, and there are LAM-epitopes more robust than others to activate the immune response. Recently, one study evaluated monoclonal antibodies that recognize different LAM epitopes in the urine of TB patients, and it was suggested that the MoAb1 antibody could be used for TBA detection in pediatric and extrapulmonary disease using nanocage technology strategy (Magni et al., 2020). However, a multicenter study is necessary to analyze the LAM-epitope quantification in order to validate that the delivered LAM-epitopes are similar among the members of each TB group.

Thus, although the LAM-epitope spectrum is a not yet clarified issue, a study of DNA sequences of anti-LAM antibodies has been recently carried out, and the analysis shows a high divergence among the location of complementarity determining regions. Few antibodies can recognize LAM of mycobacterial clinical isolates (Yan et al., 2020). New methods are necessary to increase the antibodies’ avidity, as for the use of anti-LAM-magnetic nanoparticle-conjugates immunoassay platform to concentrate the antigen has proven to increase 50 to 100-times the test sensitivity compared to conventional ELISA (Hamasur et al., 2015). Similarly, the use of nanotechnology has served in the application of a copper complex dye within a hydrogel nanocage that captures LAM with remarkably high affinity. Authors reported that this method increases 100–1,000-times the sensitivity to LAM detection in pre-treatment urine (Paris et al., 2017).

Interestingly, the commercial urinary LAM antigen testing has optimal recognition ranging from 0.05 to 10 μg of LAM/ml, and the specificity decreases at higher or lower LAM concentrations (García et al., 2019). Interference with the test sensitivity due to large amounts of LAM in urine could be explained by the “shielding effect,” which is defined as the interference between LAM-epitopes and anti-LAM antibodies by the presence of other biomolecules (Correia-Neves et al., 2019). Two approaches have been proposed to solve this problem: (1) urine enzymatic treatment, to break the bonds of diverse structures such as lipids and proteins, and (2) urinary lipid extraction, by using organic solvents to clean and homogenize the urinary sample. Both methods have been utilized as pre-treatment procedures for human urine before LAM testing, and it has shown an improvement since the test was 10 times more sensitive compared to no pre-treatment (García et al., 2019).

The gas chromatography/mass spectrometer (GC/MS) method is an alternative technique for LAM detection. Urine LAM was detected in a range of 3 to 28 ng/ml; the sensitivity and specificity of this technique was compared to the classic ELISA. Authors concluded that the GC/MS method is a viable alternative to evaluate urine LAM. They suggested that serum could be an additional reservoir for developing a POC test based on LAM (Amin et al., 2018). Although GC/MS method is optimal for quantifying proteins, the principle of adsorption of hydrophobic interaction chromatography (HIC) that separates molecules based on their hydrophobicity over high-salt separation medium like sepharose is also useful. This medium limits the solvation process of LAM and hydrophobic regions that become exposed are adsorbed by the media. This methodological advance could be useful to POC test devices and could be the solution against matrix components that interfere with the assay of LAM. The electrochemiluminescence platform (ECL) is another technique that has provided better results in the early diagnosis of TB and in the development of new platforms for point-of-care TB assay based on LAM detection, which is already contemplated in multicenter studies (Sigal et al., 2018; Broger et al., 2019b).

Dahiya et al. (2019) introduced the immuno-polymerase chain reaction (I-PCR) for detection of ultralow urinary LAM. It is based on a detection-specific antibody with a reporter DNA-antibody, and it is examined by electrophoresis or real-time analysis of PCR. I-PCR showed increased sensitivity for LAM detection. This technique can detect LAM levels 106-fold lower than conventional ELISA. It means that I-PCR detects as low as 1 fg/ml (sensitivity of 74% and specificity of 91.5%). This test has emerged as an excellent tool for LAM-testing in humans since the WHO has specified that diagnostic TB tests should have a minimum specificity of 98% and general sensitivity >65%.

Although several methods have the potential to develop more sensitive and specific tests to detect LAM in urine, intriguingly there is also evidence of circulating LAM, which can be used as a serum biomarker for TB diagnostic. Furthermore, there are critical insights of urinary LAM as a phenomenon indicative of renal disseminated TB, and the assays could be focused on identifying early stages of renal dissemination as well, such as the ones shown in the Xpert MTB/RIF clinic assay for extrapulmonary TB in urine (Kohli et al., 2018; LaCourse et al., 2018; Schutz et al., 2019).

The implementation of urine LAM quantification as a point-of-care TB assay is still far because there are several inconveniencies focusing mainly on the sensitivity, costs, and unclear results. Table 1 shows an analysis of the current assays for urine LAM quantification. It shows that the opportunities for developing and improving the sensitivity and specificity of urine LAM tests are still open.

**Table 1:** Advances in urine LAM assay for TB diagnosis.

Test or technique (antigen/antibody)	Detection limit	HIV+			HIV−			Product development detail
		^*^Sensitivity	^*^Specificity	References	^*^Sensitivity	^*^Specificity	Reference	
ALERE DETERMINE TB (pAb)	NS	Related to CD4 cell count (33.3 to 57.15)	>85	Bjerrum et al., 2019; Cresswell et al., 2020; Tlali et al., 2020	10.8 (6.3 to 18.0)	92.3 (88.5 to 95.0)	Thit et al., 2017; Broger et al., 2020	Access
Fujifilm SILVAMP TB (MTX/MoAb1)		70.4 (53.0 to 83.1)	90.8 (86.0 to 94.4)	Broger et al., 2019a	53.2 (43.9 to 62.1)	98.9 (96.7 to 99.6)	Broger et al., 2020	Validation
GC/MS (ManLAM)	3–28 ng/ml	>99.0	>84.0	Amin et al., 2018	75 (52.9 to 89.4)	92.8 (75 to 98.7)	Amin et al., 2018	Feasibility study
GC/MS (D-Arabinose)	<10 ng/ml	65.5 (52.0 to 79.0)	>83.0		>82.0	>83.0		
Nanocages technology (MTX/MoAb1)	80 pg/ml	>90.0	>70.0	Magni et al., 2020	>90.0	>70.0	Magni et al., 2020	
ECL (S4-20)	11 pg/ml	100.0 (87.0 to 100.0)	93.0 (70.0 to 100.0)	Sigal et al., 2018; Broger et al., 2019a	80.0 (55.0 to 93.0)	100.0 (84.0 to 100.0)	Sigal et al., 2018; Broger et al., 2019a	Development
ECL (MTX/MoAb1)	1.6 pg/ml	NR			66.7 (57.5 to 74.7)	98.1 (95.6 to 99.2)	Broger et al., 2020	
Immuno-PCR (pAb)	1 fg/ml	NR			75.0 (52.9 to 89.4)	92.8 (75 to 98.7)	Dahiya et al., 2019	Feasibility study
P-FAB (ManLAM/IgG and IgM)	1 fg/ml	NR			(43.0 to 78.0)	NR	Divagar et al., 2020	

NR, Non reported; pAb, polyclonal antibody; ECL, sensitive electrochemiluminescence, immunoassays; GC/MS, gas chromatography/mass spectrometer; P-FAB, plasmonic fiber optic absorbance biosensor; NS, non-determined.

* Percentage.

### Advances in the Use of Serum LAM Assay to TB Diagnosis

An alternative to urine LAM testing is the possibility to detect LAM in peripheral blood. Sada et al. (1992) proposed LAM detection as an antigen measurable in blood, by ELISA, whose preliminary results showed a minimum sensitivity of 57% and specificity of 100%. This alternative had a better predictive value for early diagnosis of TB. New platforms, such as electrochemiluminescence immunoassays and single-molecule array (SIMOA), can also be helpful for the development of TB diagnostic tools. These techniques have achieved very good sensitivity because they detect the levels using a femtogram. However, sensitivity and specificity are affected in HIV-negative individuals, and reports have demonstrated that LAM is efficiently detected in TB patients with HIV co-infection. HIV-negative patients showed a loss of both sensitivity and specificity that was inversely proportional to the lymphocyte counts; it means that higher CD4^+^ T cell count correlated with lesser test sensitivity (Broger et al., 2019b; Brock et al., 2020).

Studies of matrix effects have shown that the blood sample pretreatment could improve the sensitivity for LAM detection, but some problems remain, such as cross-reactions, low adsorption, immunocomplex formation, or the low LAM concentration in serum. Laurentius et al. (2017) showed the importance of LAM-protein interactions as a primary interference to detect LAM in blood. Authors suggested that treatment with acid (HClO_4_), heat, or methanol is efficient for protein denaturation in human serum, and it is useful to improve the sensitivity of the ELISA test.

Thus, due to the blood LAM-ELISA’s lower sensitivity and reduced association with host clinical factors compared to the urine-LAM test, it has been difficult to establish this technique as a new diagnostic tool (Wood et al., 2012). Studies have highlighted an essential role of HDL binding to amphiphilic pathogen biomarkers like lipoteichoic acid (LTA) and lipopolysaccharide (LPS), causing a reduction in the specificity and sensitivity of any diagnostic test (Sakamuri et al., 2013; Kubicek-Sutherland et al., 2019b). Thus, the presence of LAM-HDL or LAM-proteins complexes in blood is also an interfering-factor for LAM to be used as a biomarker in TB diagnosis. On the other hand, some reports suggest that LPS-HDL complexes are advantageous because they are distributed throughout host lipoproteins in serum, which contributes to reducing the endotoxic activities *in vivo*. This is a classic regulatory mechanism mediated by TLR-LPS signaling and CREBH transcription factor that directly activates the expression of the gene encoding apolipoprotein A4 (ApoA4; Baumberger et al., 1991; Dandekar et al., 2016).

The genus *Mycobacterium* is a source of several amphipathic molecules. For instance, *Mycobacterium ulcerans* produces the toxin Mycolactone, which seizes soluble lipophilic molecules in human serum as a pathogenic mechanism to escape from antibody recognition (Kubicek-Sutherland et al., 2019a). Furthermore, it is essential to note that LAM can move inside of bacterial vesicles; thus, this is probably one of the reasons why LAM avoids antibody recognition (Athman et al., 2017). Observations suggest that during the logarithmic phase of pathogenic bacterial growth, many amphipathic molecules are embedded in membrane vesicles which are posteriorly released (Shiraishi et al., 2018).

Little has been explored about the consequences of structural modifications in LAM before being released. Reports suggest that a glycosylation system or addition/breaking off of molecules is critical to delivering amphipathic molecules, like LTA from *Clostridium perfringens* or LTA from *Staphylococcus aureus* (Kho and Meredith, 2018; Wenzel et al., 2020). Mtb is capable of substituting α-(1→2)-Manp-linked residues with α-(1→4)-linked methylthio-D-xylose (MTX) residues, and modifying the branches of arabinofuranosides and mannose-capped versions, decreasing recognition of antibodies that are currently used in diagnostic methods based on immunoassays (Taghikhani and Moukhah, 2016; Angala et al., 2017; Choudhary et al., 2018). It is crucial to consider the association with host lipoprotein carriers and characterization of the antigenic heterogeneity in order to get a clinical diagnostic tool and facilitate the efficiency of TB detection.

In the same way, experimental evidence also indicates that LAM’s amphipathic properties allow its binding to membrane cells. This was observed through the use of specific fluorescent antibody labelling combined with confocal microscopy scanning (Sande et al., 2016). According to our hypothesis, it will open up new possibilities for immunoassay detection strategies for Mtb (Erokhina et al., 2019). Not only would detection of histological specimens be possible, but also the detection of LAM from peripheral blood.

Hur et al. (2015) found that levels of LAM-specific IgG could be used as a point-of-care test because it is rapid and economic. More interestingly, the serum IgG responses to Mtb antigens were different between _A_TB and _L_TB. This suggests that it is an excellent alternative to differentiate the TB spectrum. A limiting factor of this serodiagnostic test is that gender could modify the specificity, increasing the false-positive result rate. In this context, the chemical characterization of LAM based on GC/MS assay has the potential to solve the false-positive result problem (Amin et al., 2018). Another possibility to solve the limitations of antibody-based techniques is the combined use of techniques using gold nanoparticle-based on surface-enhanced Raman scattering (SERS) detection. This method increases the sensitivity and specificity of LAM detection in non-HIV TB infected patients (Crawford et al., 2017). However, it should be considered that the development of two techniques in parallel increases the cost of the diagnosis. A summary of the main tests of LAM quantification in blood is shown in Table 2.

**Table 2:** Advances in serum LAM assay for TB diagnosis.

Test or technique (antigen/antibody)	Detection limit	HIV+			HIV−			Product development detail
		^*^Sensitivity	^*^Specificity	Reference	^*^Sensitivity	^*^Specificity	References	
LF-LAM (ManLAM)	21 pg/ml	NR			~88.7	~90.0	Youssef et al., 2016	Feasibility study
ECL (S4-20)	11 pg/ml	>48.0	100.0 (80.0 to 100.0)	Broger et al., 2019b	NS	100.0 (84.0 to 100.0)	Broger et al., 2019b	
ECL (FIND 28)	6 pg/ml	>76.0	100.0 (80.0 to 100.0)		>20	100.0 (84.0 to 100.0)		
SIMOA (FIND 28/A194-01)	2.3 pg/ml	37.0 (27.0 to 48.0)	100.0	Brock et al., 2020	20.0 (8.0 to 37.0)	>98.0	Brock et al., 2020	
SERS-based immunoassay (ManLAM/CS906.7)	2 ng/ml	NR			>85.0	>97.9	Crawford et al., 2017	
ELISA (Man1Ara4/CS40)	1 ng/ml	NR			>99.0	>92.0	Amin et al., 2018	
ELISA (LAM-specific IgG)	NR	NR			~65.5	~70.4	Hur et al., 2015	

NR, Non reported; ECL, sensitive electrochemiluminescence; P, pretreatment in samples; SIMOA, single-molecule array; SERS, surface-enhanced Raman scattering; LF, lateral flow; ND: non-determined.

* Percentage.

## Future Development and Limitations for Lam Detection

Although the sensitivity and specificity of urine LAM test are currently improving, studies have shown that the clinical profile of a TB patient interferes with the diagnostic assays, as also happens in the interferon-gamma release assays (Rao et al., 2021). Historically, the development of diagnostic methodologies based on ELISA opened the door to first-generation LAM tests. However, several limitations are still present in the development of techniques, including low sensitivity in immunosuppressed patients, low specificity of monoclonal antibodies, matrix effect of the analyzed sample, continuous remodeling of LAM structure, and consequently accessibility to epitopes.

The next-generation LAM tests should have characteristics such as being easy-to-use, rapid, economic, and being a non-sputum point-of-care assay test, in order to satisfy the WHO high priority requirements (Bulterys et al., 2019). Given the current situation, we consider that focusing our efforts on improving the diagnostic tools based on the identification and quantification of LAM in urine or plasma is imperative, as it could have several characteristics to become a gold-standard diagnostic test. For instance, LAM is localized in an accessible compartment (urine or blood), and optimal development of specific antibodies will be helpful to develop cheaper tests. LAM quantification does not require long periods to yield a result, as the current Mtb culture do.

Several questions still need to be answered to establish the LAM quantification as a point-of-care assay test. We suggest that the use of chemical digestion has improved the sensitivity and specificity of diagnostic tests based on LAM, and reports have suggested that the enzymatic digestion favors the display of the classical terminal end of LAM structure (De et al., 2020). It could also be beneficial to identify the best epitopes to activate a strong immune response, and consequently to obtain better monoclonal antibodies that, in turn, would be helpful to improve the sensitivity and specificity of the LAM-based tests.

Other biomolecules, both from host and Mtb, could be used as biomarkers, but the main disadvantage is that these are not specific for TB, although they have been correlated with clinical status (Eribo et al., 2020). Specifically, 44 antigens of Mtb of protein origin, such as amidohydrolase, citrate synthase 1, and ESX-1 secretion system protein eccB, have been proposed as candidates for diagnosis in TB patients (Kim et al., 2016). In this context, and even with the limitations mentioned above, LAM is still the best strategy for the development of a diagnostic test for TB patients.

## Concluding Remarks

Early diagnosis of Mtb infection represents an opportunity to initiate timely treatment against the infection, and consequently to avoid Mtb spread in the community. Still, many people worldwide are infected with Mtb during their whole life as _L_TB patients; these patients do not display clinical symptoms. Moreover, they have a high potential to spread the infection, although _A_TB patients have a higher mortality rate than _L_TB patients.

Currently, it is necessary to perform several tests to have a TB diagnosis. Despite the availability of new diagnostic techniques, the smear test and the culture of the microorganism are the gold-standard tests, but these are expensive and deliver late diagnoses. _L_TB diagnosis is also complicated; for several years, PPD test has been used to identify _L_TB patients. However, the result must be interpreted carefully due to several factors that influence a positive result.

As was discussed above, the current proposed methodological strategies show that further efforts are necessary in order to establish LAM as an optimal point-of-care test for TB diagnosis. To date, it is necessary to improve some disadvantages such as: (1) technique sensitivity, (2) the interaction of LAM with cells or soluble molecules, and (3) the structural changes of LAM. However, LAM also provides advantages such as: (1) being a specific molecule of *Mycobacterium* infection, (2) having an accessible site of localisation (blood and urine), (3) its immunoregulatory functions, and (4) being a potent activator of the immune response. This review shows the relevance of LAM to establish a new point-of-care test. Interestingly, LAM could also be useful to differentiate the broad spectrum of TB diagnosis.

## Author Contributions

JF wrote the manuscript and made the figures. JC provided a critical reading of the article. LC-G conceived and designed the original idea, and supervised the manuscript. All authors contributed to the article and approved the submitted version.

### Conflict of Interest

The authors declare that the research was conducted in the absence of any commercial or financial relationships that could be construed as a potential conflict of interest.
